# General considerations for research with vulnerable populations: ten lessons for success

**DOI:** 10.1186/s40352-014-0013-z

**Published:** 2015-01-21

**Authors:** Camille R Quinn

**Affiliations:** Department of Psychiatry, School of Medicine & Dentistry, 300 Crittenden Boulevard Box PSYCH, Rochester, NY 14642 USA

## Abstract

**Background:**

This paper offers practical insights for researchers who conduct studies with vulnerable populations, including those using secondary data sources from government entities.

**Methods/Design:**

The paper is based on the approval process to secure secondary government data from a Midwestern county juvenile probation department and the state courts for the author’s dissertation study.

**Discussion:**

This paper proposes general considerations and ten lessons learned to successfully conduct research with vulnerable populations and/or their information.

Youth involved in the juvenile justice system comprise a vulnerable population based on their age and because of their system involvement. Scholars have identified a profile of vulnerable youth that is made up of primarily poor, African American males (Foster et al. 2005), and risk factors associated with delinquency (Fraser et al. 1999; Werner 2000). Consequently, future research must continue to elucidate ways to address the complex and multi-faceted needs of vulnerable populations, especially those involved in the juvenile justice system. One of the key issues facing researchers studying justice involved youth, is their designation as a vulnerable population, and how that impacts the researchers’ ability to include them as subjects in their studies. Consequently, researchers whose studies include vulnerable populations need to understand the specific issues related to conducting research with them.

Researchers face many challenges in conducting research, especially when vulnerable populations are involved. The Department of Health and Human Services (DHHS) federal regulations recognize certain categories of individuals as ‘vulnerable,’ so “they need to be accorded special protections to make sure that researchers do not take advantage of them” (Coleman, Menikoff, Goldner, and Dubler 2005). Also, the federal government regulates human subjects research with institutional review boards (IRBs) at academic institutions that conduct research studies. Among other things, the IRB wants to ensure that researchers maintain privacy and confidentiality of subjects (Schwenzer 2008) or the subjects’ information. Achieving IRB approval for studies is a complex and time-consuming process, and studying vulnerable populations, such as youth in the juvenile justice system, makes the process even more difficult. Moreover, the use of government information about vulnerable youth requires the approval of government entities as well as IRBs.

## Background

Human ethics and research committees act as research gatekeepers, and since many research activities involve multiple applications and approvals, the process can significantly delay the start and completion of a project (McCauley-Elson et al. 2009). Consequently, it is critical for researchers to understand the ethical principles outlined in the Belmont Report, especially when studying vulnerable populations. The Belmont Report is a useful reference for highlighting important considerations about conducting research with vulnerable populations including justice-involved youth. The report, prepared by the National Commission for the Protection of Human Subjects of Biomedical and Behavioral Research, is a declaration on the ethical principles overseeing human experimentation (Public Health Service 1979). The report continues to articulate enduring normative standards regarding research practices with vulnerable populations even though it was originally issued in 1979 (Dresser 1996).

Prisoners are a particularly vulnerable group who have historically been included as research subjects but have also been exploited by investigators in pursuit of useful solutions to complex research problems (Gostin, Vanchieri, and Pope 2007; Hornblum 1997, 1998; Mitford 1974). Moreover, youth involved in the juvenile justice system are an especially vulnerable group of research participants who are considered to be less able to defend themselves, and require special protection from possible abuse, exploitation, or discrimination by others in positions of power (Rose, and Pietri 2002).

## Methods/Design

The method for this paper was framed using the three basic principles of the Belmont Report: 1) Respect for Persons, etc.

The three basic ethical principles in the Belmont Report: 1) Respect for Persons, 2) Beneficence, and 3) Justice provide general judgments to justify and guide research involving human subjects. The aim of this paper is to provide future scholars and graduate assistants with a guide to conducting research with vulnerable populations, especially those involved in the juvenile justice system. The ten lessons were devised based on the author’s experience securing the government administrative data to complete a dissertation using secondary analysis.

The issues associated transcend juvenile delinquency as incarceration impacts all vulnerable populations including justice involved women. Specifically, incarceration impacts vulnerable populations based on their race, gender, age, and socio-economic status as many of them may be impoverished. Consequently, it is prudent to recognize and reinforce the specific issues that are associated with conducting research with these groups.

## Ten lessons to successfully conduct research with vulnerable populations using the Belmont report

### Respect for persons

The Belmont Report defines respect for persons in research as:

…honoring the choices of subjects capable of exercising autonomy and protecting subjects incapable of making their own decisions. Ethical human experimentation preserves respect for the subjects whose participation is essential to the humanitarian mission of biomedical and social science research. (Dresser 1996)

In the context of studying detained or juvenile offenders, providing significance to these simple instructions is a complex undertaking.

#### Lesson 1: There’s no ‘simple’ way to conduct studies with vulnerable populations, but make every attempt to be prudent

The inclusion of vulnerable subjects in a study, especially youth involved in the justice system, significantly adds to the difficulty. This is because justice involved youth may not be capable of exercising autonomy to make their own choices, so they require protection. Their inclusion adds to the complexity that a researcher must address in their research plan. Also, collecting data directly from justice involved populations may not even be an option depending upon the geographic location. So, it’s critical for the researcher to have a clear understanding about the feasibility of the study and the greater likelihood of what is needed to successfully complete it. For example, many researchers suggest using secondary data for research studies because of its accessibility, convenience, and reduced costs in time, money, and inconvenience to participants (Vartanian 2010). However, these issues become more challenging when juvenile offenders are included given their involvement in government systems that must also approve the research project. Specifically, *parens partriae*, “the principle that the state must care for those who cannot take care of themselves” is applicable to juvenile offenders (Campbell 1991, p. 769; Snyder, and Sickmund 2006). The researcher has the task of securing approval from their local IRB, as well as the government entity(ies) to use the data for empirical investigation. The research plan must clearly articulate the steps that will be taken to protect the youths’ information, identity and confidentiality.

#### Lesson 2: Information, like people, requires protection

The Belmont Report contains a requirement to protect those with diminished autonomy, including those in need of extensive protection, such as juvenile offenders. Yet, what must be considered when the information about the juveniles is the focus of the study versus the juvenile offenders themselves? In this case, the youth are not exercising autonomy because those acting on their behalf are. Consequently, the researcher must bear in mind that the information they want is tied to those (vulnerable) individuals. Unlike other large-scale publicly available datasets, the information used for the author’s dissertation study was not collected for research purposes; it is routinely collected by juvenile justice personnel to conduct case planning with the youth and their families. Also, the study required that unique identifiers be included in the data files so they could be merged to conduct the analysis. The proprietors of the data had a primary concern about the author’s procedures to ensure protection of the individuals’ information. Consequently, data security procedures were devised to assuage these concerns (these procedures are discussed in Lesson 3).

Specifically, one datafile came from local responses on the Youth Assessment and Screening Instrument (YASI), which assesses risk, need, and protective factors for youth on probation. Also, probation officers completed the YASI to develop case planning services for the youth. For each case, they score the YASI following multiple semi-structured interviews with the youth, parents or an alternative legal guardian. Interview-based data are supplemented with a systematic review of collateral sources including police files, probation records, as well as school and mental health reports (Orbis Partners, Inc. 2007). The information collected with this instrument constituted the basis for the dataset used in the author’s dissertation study. Use of the YASI results in a score that is used for risk classification (M. Lewis, personal communication, October 10, 2012) and the scores help probation officers develop the case plans for youth. The single YASI score is not used in this study, but all of the individual measures of risk and protective factors are to create variables that were analyzed in the study. The YASI “can be used in juvenile probation, detention, day reporting, youth services, schools, police diversion and other settings with a requirement to assess risk of negative outcomes and identify service needs” (K. Hickey, personal communication, December 15, 2011; Orbis Partners, Inc. 2007). Probation officers enter YASI information into the Caseworks database while the YASI files are maintained by a private entity (subcontractor with the Administrative Office of the Courts) and their Director of Research created a codebook for the datafile.

The other data used in the dissertation study included an indicator for recidivism that was derived from the probation department management information system. Data from 2011 to 2013 were used in this study. This file included entries for all court orders that occurred after an initial court finding leading to probation. In addition, demographic information was included: age, ethnicity, and additional unique identifiers (probation ID#, name and birthdate) needed to merge the YASI and recidivism datafiles into a unique dataset for the study. Probation officers also enter the probation and recidivism information into the computer system and the Office of the Chief Judge and the probation department who manage the files. The merge was conducted by a statistics and database consultant and comprised a series of steps to link youth in the YASI and recidivism files using the probation ID#, petition ID, last name, first name, and DOB. First, the files were linked using ID#, a unique identifier assigned to each youth. The probation ID# is generated by the state's attorney's office for each youth. While the probation ID# was ideal, it was not available for each youth’s record in the YASI file. Therefore, a merge strategy was employed to create a recidivism file using probation ID#. For those records that did not find a match using the probation ID#, a second merge matched records using a petition ID. The County Clerk’s Office assigns petition IDs for any case the youth has and some may have several petitions ID numbers but only one probation ID#. A final merge matched records on first name, last name, and date of birth. Some of the records from the recidivism file did not merge with the YASI file either because (1) the youth didn’t recidivate or (2) the youth recidivated but the merge failed, 3) records were in the YASI file and not in the recidivism file, and 4) records in the recidivism file and couldn’t find a match in the YASI files. Several checks were conducted to identify the files that did not successfully merge with the YASI file and 15 records were found and manually entered. The inability to merge all of the records resulted in an error rate of 23%. After the records were successfully merged, a new database was created and it was deidentified by stripping the youth names, birthdates, and ID numbers from the dataset. All of these steps were taken to ensure the privacy and confidentiality of the youth information that was used in the author’s study.

### Beneficence

Promoting human welfare is the focus of the beneficence principle. The Belmont Report suggests two moral duties in a research context: 1) avoid harm, and 2) maximize possible benefits and minimize possible harms. Current federal policy includes additional requirements meant to reduce the experimental risk to which children, prisoners, and fetuses are exposed (Dresser 1996).

#### Lesson 3: Procedures for data security need to be thorough even if they didn’t exist previously

The initial response from the convened review from the author’s university IRB included several modifications including a request for the contract with a state court’s subcontractor so they could review the data security procedures that should be included. However, the subcontractor did not have specific procedures for third parties and neither did the university. Therefore, the author consulted with numerous IT experts at the university including the College of Social Work IT department and the University Information Technology Officer to create the appropriate security procedures.

Consistent with the Belmont Report, procedures to reduce risk and the potential risk of harm to subjects in this study was critical even though this study only involved administrative data with minimal risks. The process to collect information for vulnerable populations had to be completed in conjunction with existing protocols of governing entities and this included the data security measures. All the data use agreements and the IRB applications and appendices reflected the specific data security procedures to ensure privacy and confidentiality of the data since it included the identifiers. The greatest risk in the author’s study involved the potential for a breach of privacy and confidentiality of the youth by sending the data files with the identifiers that were required to merge them. Some of the procedures included having a dedicated computer in a locked office on campus. Also, this computer had to undergo software upgrades including the installation of firewall, antivirus, secure file transfer and encryption software, so that all data manipulation took place on the encrypted computer. In addition, individuals at each organization that would transfer the data had to use secure file transfer software as well. Representatives from each organization were assigned a university ID and password (registered to the author’s faculty sponsor for site access) to transfer the datasets to the secure site. After the data files were secured and successfully merged by the statistics and database consultant, the identifiers were stripped resulting in a merged deidentified dataset. With these procedures, the risk of a confidentiality and privacy breach was addressed to the greatest extent possible.

#### Lesson 4: Trailblazing for your study requires tremendous effort but could yield significant rewards

Devising a study that has not been done before is a herculean task and pursuing one with vulnerable populations only adds to the potential for scrutiny. The ultimate focus of the beneficence principle is to not do harm and maximize benefits while minimizing harm to the subjects. Since the information in this study had never been used for empirical investigation, the author had to lead the effort in bringing the study to fruition. Numerous issues had to be addressed.

#### Lesson 5: Findings could be useful without direct benefits

It is understood that research burdens should not be imposed on historically disadvantaged populations including those involved with the justice system. However, to abandon research on these conditions alone would suggest that their issues warrant less attention than other research topics (Dresser 1996). There were no direct benefits to the subjects in this study as their information was the focus not the subjects themselves. Yet, the findings may inform practitioners’ understandings about this sample of youth on probation. For example, highlighting the unmet needs of youth on probation that are associated with their risk factors could provide practitioners with indicators for service provision to reduce their delinquent behavior (Abram, Teplin, McClelland, and Dulcan 2003; Lipsey and Cullen 2007; Lipsey, Wilson, and Cothern 2000; Nissen 2006; Teplin et al. 2002).

### Justice

The concept of justice in research rests on the duty of the researcher to fairly distribute the benefits. In addition, research burdens should not be disproportionately imposed on historically disadvantaged persons, a category that includes juvenile offenders (Dresser 1996). Specifically, the selection of “human subjects needs to be scrutinized to ensure that certain classes of people are not being systematically selected because of their availability, their compromised position, or their manipulability, rather than for reasons directly related to the problem being studied” (Public Health Service 1979).

#### Lesson 6: Remember the “so what” factor about your study

It is helpful to have a prepared “elevator speech” about your study and it should include: who, what, why and how. This is especially important when vulnerable populations are the subjects in the study as this information will have to be communicated often and to multiple stakeholders. The more succinct your description of the study is, the better; in particular, the “why” part should be solid, given the potential scrutiny associated with research on vulnerable populations. For example, research has often focused on the risks associated with juvenile delinquency and recidivism and this study focused on both risk and protective factors that youth possess. In addition, the study addressed a major gap as the majority of research has been conducted on boys and detained and incarcerated youth, so the focus on probation and girls was unique. Also, having discussions with probation administrators in which they described the need for this investigation reinforced the author’s ability to convey the importance of the study to others.

#### Lesson 7: Bring stakeholders to the table to obtain buy-in

For research to contribute to addressing major social issues, collaborations between researchers and practitioners (including government staff, administrators and policy makers, and community advocates) are increasingly being seen as vital. Even in the case of this study that used juvenile offenders’ information, their selection was directly related to the problem but government agencies still scrutinized the research study given the compromised position of the youth. Academic-practitioner collaborations may be the best approach to assuage some of the challenges associated with research on vulnerable populations. Specific examples include framing research questions in a way that will be meaningful to practitioners, gaining access to field research sites or information needed for the study, and interpreting results accurately within their professional context. Given the potential benefit of academic-practitioner collaborations as well as potential difficulties involved in joint work between individuals with different perspectives and priorities (Bartunek and Louis 1996; Nyden, and Wiewel 1992), it is important to note what makes collaborations successful.

Collecting data from government agencies can be challenging, so having relationships with appropriate contacts can also make a difference in the approval process. Identifying key people early on and throughout the process will enhance the researcher’s ability to think about the nuances of the study. The author started with an administrator in juvenile probation and he eventually joined the research team. He directed the author to the state courts and a state subcontractor that houses the data needed for the study.

Some research notes some important collaborative team requirements, which concern attitudes and motivation including the creation of trust, the absence of hidden agendas, as well as the presence of mutual respect among group members (Easterby-Smith and Malina 1999; Jassawalla, and Sashittal 1998). Furthermore, trust has been found to come from the belief that the collaboration will be long-lasting (Jassawalla, and Sashittal 1998). The author’s experience building collaborations with representatives from government entities to secure information for the study was built on trust and transparency. First, the author developed an awareness of the complex web of interconnections: university, probation, courts, and a private entity affiliated with the courts, etc., and the need to attend to each one in a unique manner. The importance of knowing the key players at each organization cannot be overemphasized, especially building and nurturing relationships with them. It took many months of discussions with county, state and other representatives before gaining a clearer idea of what was needed to proceed with the study. Having a relationship with one or more of these individuals is critical because government employees understand the bureaucracy that surrounds their work, and their intimate knowledge of “how things are done” could make or break the IRB approval process, as well as the success of the study. Also, government employees must follow regulations that operate regardless of the research needs and/or expectations. For example, it took the author nearly two years to develop a relationship with a key contact at the state courts. Ultimately, the best course of action was to be persistent, identify the best way to communicate with them (e-mail, phone or fax), and follow up with them relentlessly. Staying mindful of this increased the likelihood of their assistance.

#### Lesson 8: Bear in mind the significance of the population you’re studying

The significance of the study is based upon the researcher’s ability to demonstrate that the study is meaningful academically and practically, which will make it easier to secure support for the study and the data. So the researcher must remain clear about why this population is needed to investigate this specific research question. This clarity will serve the researcher well especially during the IRB approval process. One colleague even suggested securing a public dataset given the lengthy approval process. However, the author was clear about the focus on youth on probation, and the special focus on girls. Specifically, research on female offenders has been overshadowed by research on male offenders even though girls are the fastest growing group of offenders (American Bar Association [ABA] National Bar Association [NBA], 2001). This is the case even though most adolescent female offenders are not arrested and/or detained because of violent offenses (Chesney-Lind, and Jones 2010). Also, girls have been recipients of a special and discriminatory form of justice since the inception of a separate system of justice for youth (Chesney-Lind 1973; Schlossman, and Wallach 1987). “With some exceptions, extensive recent scholarship focusing on gender and crime has tended to concentrate on women, not on girls” (Zahn et al. 2009). Consequently, there has been a growing recognition that a significant number of young women and girls engage in aggressive and antisocial behaviors including traditionally male anti-social behaviors like truancy, delinquency and substance abuse (Kann et al. 1997; Poe-Yamagata and Butts 1996; Schaffner 1998). Scholars have begun to note that girls have negative interpersonal relationships (Ehrensaft 2005), histories of abuse, mental disorders, and trauma (Teplin et al. 2002). Research that highlights the needs of vulnerable populations and directly relates to the problem of juvenile recidivism could further efforts to reduce the risk of recidivism by addressing their underlying needs (Farrell et al. 2011). Consequently, the selection of this vulnerable population was prudent and directly related to the problem of juvenile delinquency.

#### Lesson 9: Do exceptional work and share it with stakeholders/constituents

Completing a research project from start to finish is a daunting task and there is really no room for error. Moreover, errors can be costly in terms of time and effort and this could have dramatic effects on a study with vulnerable subjects as participants. For example, the process of securing IRB approval was not only lengthy, but there were many unknowns because no study had been conducted with this information from juvenile probation before. Numerous tasks had to be completed prior to submitting the final IRB application, including multiple data use agreements, IRB approval from the county bureau of health services (which required additional human subjects training certification for all parties involved with the study), and the creation of data security procedures. The author provided regular updates via meetings (as needed), e-mail with written communication and phone conferences to the research team and all the constituents so everyone was informed of the study’s status.

The status updates continued throughout the project, including when the data files were received, analyses completed, and once all the results were compiled. Part of the agreement with probation and the state court (and their subcontractor) was that the author would provide each entity with a final report about the study, which was submitted in May, 2014. It was also understood that the author would not conduct any presentations on the findings from the study until the report was disseminated to the constituents.

#### Lesson 10: Persistence and principled: knowing your work matters

Securing and using government data on vulnerable populations can be a complex process, so establish a solid research plan and commit to it. This can be tricky because it may not be easy to anticipate all that is involved in a study like this, especially when the subjects, or information about the subjects, are court involved. That is why it is so important to spend time in the beginning brainstorming the significance of including this population and the steps needed to secure the data. This process will be important to the researcher throughout the study, especially if the need to entertain other data options arises. Aside from the actual IRB review process, there will be procedures that must be followed to receive the data, as well as procedures required to handle the data once it has been received. Each step will require a portion of time, so try to map it all out and include extra time to meet these deadlines. The author’s IRB approval process took nine months, which did not include the countless conversations with government staff, onsite meetings, or visits to hand deliver or pick up time sensitive materials. The time spent obtaining this information and building relationships added up quickly, so there should always be identified tasks to focus on if and when lulls in the process arise.

Despite the best plans, issues arose that seemed impossible to address, but energy must always be redirected to the purpose and intent of the study. For example, the author’s data analysis plan was set back by two months because of delays in getting the data from the private subcontractor. Once the data files were received the initial plan to create and code variables was also protracted based on the need to gain familiarity with the data files. The author had a statistics and database consultant to create the database and analytical datafile, as well as strip the identifiers from the dataset. A major issue with the recidivism data was that the timeframe was much shorter than desired due to the timeframe in which the probation department started collecting this data. Specifically, YASI data was collected from 2001 to 2013 and the recidivism data was collected from 2009 to 2013, so the study dataset only comprised youth matched to YASI files from 2009 to 2013 as well (n = 5831). This yielded a much smaller sample of youth including those that recidivated (7%) and the local recidivism rate in this county is 17%.

## Discussion

This study was not undertaken without proper consideration for all the factors involved, especially time. Clearly, it would have taken much less time to complete this study if the author had secured a publicly available dataset, but this convenience did not outweigh the practical significance associated with the government issued data about youth on probation. The time investment was worth it as the author has completed the study and final report, which has been disseminated to all the constituents. Also, the author was invited to present the study findings to a state council meeting where continued interest was expressed in the project, which could result in favorable changes for justice involved youth in that county.
